# The development of an online database and mobile web application for the collection and analysis of hand hygiene compliance data

**DOI:** 10.1186/1753-6561-5-S6-O30

**Published:** 2011-06-29

**Authors:** PL Russo, K Heard, M Cruickshank, ML Grayson

**Affiliations:** 1 Hand Hygiene Australia, Heidlelbrg, VIC, Australia; 2 Australian Commission on Safety and Quality in Health Care, Sydney, Australia

## Introduction / objectives

Hand Hygiene Australia (HHA) commenced implementation of a National Hand Hygiene Initiative (NHHI) in 2008 into all healthcare facilities. Based on the World Health Organization “5 Moments for Hand Hygiene” Program, one of the aims is to establish a uniform system of hand hygiene (HH) compliance auditing to allow local, national and international benchmarking. HH compliance (HHC) data was collected manually on a paper form, input to a local database and then emailed to HHA. This process was time consuming, resource intensive.

## Methods

An online database (OLDB) was developed allowing both manual data input and uploading of data collected on a mobile device. Further development included a mobile web application (MWA) which provided for use of mobile devices (Smartphones, iPads etc) to collect data and direct submission into the OLDB via a web interface. The OLDB allows for instant reporting of HHC rates at a unit, hospital, state and national level, by Moment and HCW. An export capability in the Report function allows users to export into other common programs for table and chart formatting to their preference.

## Results

Development of the OLDB was completed in July 2010. In March 2011, over 360 hospitals submitted data using the OLDB. The development of the MWA was completed in February 2011, and its use is increasing.

## Conclusion

A cost effective, user friendly electronic HHC data collection and analysis process is now available for all Australian healthcare facilities. Anecdotal reports indicate that the use of the MWA and OLDB has halved the time taken to manage HHC data. This development has been vital to the sustainability of the NHHI and is potentially usable by other national programs.

## Disclosure of interest

None declared.

